# Accuracy of tympanometry cutoff points in children from Central-Western Brazil

**DOI:** 10.1590/2317-1782/e20250100en

**Published:** 2026-08-03

**Authors:** Cláudia Cruvinel Câmara, Melissa Ameloti Gomes Avelino, Paulo Sergio Sucasas da Costa, Alexandre Siqueira Guedes Coelho, Maria Claret Costa Monteiro Hadler

**Affiliations:** 1 Programa de Pós-graduação em Ciências da Saúde, Faculdade de Medicina, Universidade Federal de Goiás – UFG - Goiânia (GO), Brasil.; 2 Programa de Pós-graduação em Nutrição e Saúde, Universidade Federal de Goiás – UFG - Goiânia (GO), Brasil.

**Keywords:** Screening, Tympanometry, Otoacoustic Emissions, Tympanometric Pressure Peak, Accuracy

## Abstract

**Purpose:**

This study evaluated tympanometric cutoff points (tympanometric peak pressure and static compliance) for transient evoked otoacoustic emissions (TEOAEs) and determined their diagnostic accuracy.

**Methods:**

This analytical study, based on a randomized clinical trial, included 84 children (6–42 months; median 24 months) from public childcare centers in Brazil. Associations between cutoff points and TEOAEs were verified by Fisher’s exact test and Chi-square. Kappa assessed agreement between ears, and the correlation between tympanometric peak pressure and static compliance was analyzed by Spearman. Diagnostic accuracy was determined using ROC (Receiver Operating Characteristic) curves and confirmed with EpiDat 3.1.

**Results:**

In the 84 children, 66 right ears and 73 left ears were examined; however, 42 ears were randomly selected from each side for the final analysis. A significant correlation was found between peak pressure and static compliance. ROC analysis identified -180 daPa as the most accurate cutoff (AUC = 0.781; 95% CI: 0.656–0.905; p < 0.001). EpiDat indicated a specificity of 93.9%, a sensitivity of 55.6%, and an overall accuracy of 85.7%. Static compliance <0.2 mL showed comparable performance (specificity = 98.5%, sensitivity = 50.0%, accuracy = 88.1%).

**Conclusion:**

The cutoff point of –180 daPa combined with static compliance <0.2 mL improved screening accuracy and reduced unnecessary referrals. It provided the best balance between specificity, sensitivity, and accuracy, aligning more closely with TEOAEs results and supporting updating this tympanometric criterion in infant hearing screening.

## INTRODUCTION

The World Hearing Report estimates that by 2050, approximately 2.5 billion individuals will have hearing loss of varying degrees, highlighting the need for lifelong hearing intervention programs. Actions to evaluate and prevent hearing disorders should be conducted early, focusing on early childhood education^(1)^.

In the first three years of life, children should receive responsive care^(2)^. Although alterations in the middle ear are common and school hearing screenings can differentiate between children with and without a risk of hearing issues^(1,3)^, such screenings are not typically conducted during a child’s earliest years^(4)^.

Alterations in the middle ear can be identified by analyzing the results of transient evoked otoacoustic emissions (TEOAEs) and tympanometry. Utilizing tympanometry and TEOAEs for auditory monitoring in preschool and school-age children is a well-established practice, evidenced in numerous studies^(1,5-7)^.

Otoacoustic emissions can be used to identify changes that impair sound conduction in the cochlea^(1)^. One application of tympanometry is to estimate the normal middle ear pressure, and when it is functioning properly^(8)^, a type “A” tympanogram is the expected response^(9-11)^. Although TEOAEs and tympanometry are recommended as instruments for hearing screening to identify asymptomatic hearing loss in preschool and school children^(12)^, they must be carried out systematically in this population^(2)^.

Additionally, recognizing the tympanometric peak pressure (TPP) value most associated with TEOAE Refer is necessary, as the decision on the tympanometry cutoff point can generate different interpretations and results. Therefore, choosing the tympanometry cutoff point is essential in the referral process. Types A and B tympanograms are well defined in the literature^(9-11)^; however, those with peaks shifted to negative pressures do not always reflect effusion^(13)^.

The TPP values observed in different age groups exhibit notable variation. In the pediatric population, values below -150 daPa have been identified as indicative of an abnormal condition^(13)^. However, TPP values below -200 daPa and static compliance values below 0.2 mL are considered indicative of an abnormal condition^(1,5,6)^.

Assuming that young children are more likely to have hearing problems and that these often occur silently, the identification of these cases is essential for public health, especially for underprivileged children. Nevertheless, depending on the TPP used, this exam may generate false positives, increasing the number of unnecessary referrals for treatment. Most studies of Brazilian children in public early childhood education have used a value of <-100 daPa as a cutoff point in hearing screening. The scarcity of national studies showing the accuracy of different cutoff points in 6 to 42-month-old children justifies the need for this study with children from the Brazilian Central-West.

## METHODS

### Study design and participants

This analytical study used the baseline of a randomized clinical trial entitled “Effectiveness of micronutrient powder fortification in the prevention and treatment of micronutrient deficiency: a randomized clinical trial.” During the participant recruitment stage, children’s legal guardians were informed about the study objectives and how to participate in the study. If they agreed to participate, they signed the Free and Informed Consent Form. The participants were children age 6–42 months, attending full-time Municipal Early Childhood Education Centers (ECECs) in Goiânia, Goiás, Brazil. From 74 ECECs linked to the Health at School Program (HSP) in Goiânia, a cluster randomization process began. This excluded ECECs that had already been supplied the Infant Feeding Fortification Strategy with Powdered Micronutrients (NutriSus) sachets, were not part of the HSP, did not care for children <1 year of age, and with <7 children in age groups of interest. Thus, 22 ECECs remained, of which two were drawn from each of the five remaining health districts of a total of 10 ECECs. The initial sample comprised 84 children, 6–42 months old, attending child education centers (ECECs) linked to the HSP in Goiânia, Goiás.

The study was conducted following ethical guidelines and approved by the Ethics Committee under number [CAAE: 80541717.3.0000.5083] and Opinion Number: 3,154,008.

### Inclusion and exclusion criteria

To be included in this study, children must have participated in the matrix study, attended the selected ECECs, and be 6–42 months of age at the beginning of the study, and their parents must have signed the Free and Informed Consent Form. The exclusion criteria were low gestational weight, premature birth (<37 weeks), and twins; reported allergy to any components of the micronutrient powder sachet and/or to ferrous sulfate and folic acid; and currently being treated for anemia, malaria, human immunodeficiency virus infection, hemoglobinopathies, or hemochromatosis.

### Data collection

For data collection, a structured form prepared by the matrix project team, together with related researchers, was used. This form contained data common to project researchers. The matrix project team applied the form at each ECEC with the children’s parents or guardians before collecting the hearing screening data.

The following variables were used to characterize the sample: sex (female and male), age in months, child’s, mother’s, and father’s races (white, brown, and black), per capita family income, economic class^(14)^, daycare time, children <5 years old at home, smoking during pregnancy, breastfeeding within 1 hour, use of bottles, and previous hearing problems.

### Procedures and equipment

The school hearing screening was conducted at the ECECs, where the Nutrition team had already been. Prior to the beginning of the school hearing screening, the environments made available by the coordination of each of the 10 ECECs were selected to carry out the hearing screening. If possible, an environment as far away from the playground and classrooms as possible in the ECECs was selected. Noise was collected with a type II sound pressure level meter, Minipa MSL-1351C Sound Level Meter (Minipa, São Paulo, Brazil; manufactured in China) in the screening environments during the TEOAEs to verify possible interference with the test results. The measurements were taken in the school environment during the TEOAEs. The decibel microphone meter was placed close to the TEOAE equipment, and the dB (HL) noise levels were collected during the TEOAE test. Minimum and maximum mean noises were recorded using the dB (A) scale in slow mode.

To perform the school hearing screening, child behavior management techniques, such as distraction (telling stories and distracting with toys) and tell-show-do (explaining, demonstrating, and performing the procedure), were used to obtain the child’s cooperation during the meatoscopy, TEOAEs, and tympanometry examinations. Ludic and age-appropriate languages were used for this purpose. The school hearing screening was initiated with the child’s permission.

Meatoscopy was performed using the M(D+) Healthcare Otoscope (Medical Devices Pvt. Ltd., Sialkot, Pakistan). The TEOAEs were assessed using the Madsen AccuScreen OAE & ABR Screener (Natus Medical Inc., Denmark). Tympanometry was performed with the MAICO MA630 Tympanometer (MAICO Diagnostics GmbH, Germany), and the examinations were performed using a 266 Hz probe tone^(9)^. All equipment was used according to the manufacturer’s instructions, following standardized procedures for each test. Meatoscopy assessed the conditions of the External Acoustic Meatus and was classified as (0) when the external auditory canal was unimpeded and (1) when there was a total impediment or even a partial impediment of the external auditory canal. Only those without an impediment in the external auditory canal underwent hearing screening.

Transient Evoked Otoacoustic Emissions (TEOAEs) were recorded with Madsen® AccuScreen, configured to emit non-linear clicks at 75 dB(A) ± 5 dB on a 2cc coupler, with volume-dependent self-calibration of the ear canal. Stimuli were presented at a rate of 67 to 76 clicks per second (randomized), with an input filter between 1 and 4 kHz. The signal processing considered the weighted average of the noise and the count of significant peaks, requiring identification of eight valid peaks.

The test was considered "Pass/Clear Response" when the counter recorded ≥ 8 valid peaks in alternating directions, four above the mid-line (high frequencies) and four below (low frequencies), responses that showed sufficient Signal to Noise Ratio (SNR) within the 1–4 kHz range, with minimum detectable amplitude above the noise (accepted limit < 60 dB SPL), in addition to the presence of a green bar indicating that the probe was well positioned. Refer/No Clear Response “good” Test → conditions were adequate (artifact ≤ 20% and stability ≥ 80%), but no clear response occurred. Refer/No Clear Response “bad” → occurred when test conditions were inadequate (artifact > 20% or stability < 80%), requiring repetition after adjustment of probe fitting or noise control. “Refer” does not necessarily indicate that there is hearing loss.

The following responses were adopted: (0) “Pass,” absence of cochlear alteration, which suggests that the outer hair cells, responsible for amplifying sounds, are healthy and functioning correctly; and (1) “Refer,” indicating that the child did not pass the screening and should be referred for retesting, and if they fail again, they will be referred for further diagnostic evaluation.

To verify the “Pass” and “Refer” in the TPP suggested in the literature, the tympanogram classifications for the peaks at -100 daPa^(9-11)^, -150 daPa^(13)^, and -200 daPa^(1,2,6)^ for static compliance of ≥2 mL^(5)^ were used as references. For tympanometry, the criterion of “Pass” and “Refer” was used in the various cutoff points (-100 daPa, -150 daPa, -200 daPa, and static compliance). The type A curve was considered as “Pass,” and type B or C curves (across the different TPP cutoff points investigated), and compliance of <0.2 mL were considered as “Refer.”

The hearing screening analysis considered both the individual results of tympanometry and TEOAE, as well as their combined interpretation, with outcomes classified as ‘Pass’ or ‘Refer,’ according to predefined cutoff points. To pass the screening, the child had to pass the TEOAE and have a Type "A" tympanometric curve.

Children who presented a type A tympanometric curve but received TEOAE Refer were retested on a different day to rule out temporary factors. Those who passed the TEOAEs on the retest were considered to have normal cochlear function.

Although the study included 84 children, not all presented adequate conditions for evaluation in both ears due to factors such as cerumen, technical issues, or other limitations. To avoid side-related bias and ensure data independence, only one ear per child was included in the statistical analysis. For the children with both ears evaluated, the selection of the ear was performed randomly, alternating between the right ear (RE) and the left ear (LE). This approach resulted in a balanced distribution of 42 RE and 42 LE to minimize any potential interference related to ear side and allowed for a more accurate analysis of the diagnostic performance.

At the end of the hearing screening, the parents and guardians received the results and necessary referrals through the ECECs. Children who received Refer were referred for retesting and received appropriate guidance for each case. Finally, all family members and professionals of the ECECs were invited to a presentation that focused on guidance and care for hearing and its relationship with a healthy diet.

### Data analysis

Double-entry data scanning was used to verify consistency (Epi Info^®^ 6.04d). Data were analyzed using the Statistical Package for Social Sciences version 18.0. The significance level for all tests was 5%. The Kolmogorov-Smirnov test was employed to analyze the normality of the variables. Absolute and relative frequencies were used to characterize categorical variables. The mean and standard deviation were used for continuous variables, with a normal distribution, and the median and interquartile range were employed for variables without a normal distribution. For noise analysis, a descriptive analysis of the data was performed, and the mean minimum and maximum noise levels were verified. The Student’s t-test was conducted to investigate the extent of the mean maximum noise levels in the TEOAEs. The Fisher's exact and Chi-square tests were used to evaluate the association between the different tympanometry cutoff points and TEOAE results. The Kappa test was employed to verify the agreement of responses between the two ears in the TEOAE tests, tympanometry at -100 daPa, -150 daPa, and -200 daPa, and static compliance. Subsequently, from the 84 participants, 42 right and 42 left ears were randomly selected to form a single bank to verify the TPP accuracy. The correlation between the TPP and static compliance was analyzed using the Spearman correlation coefficient to verify the relationship, direction (positive or negative), and degree (strength: weak, medium, and strong). Next, the receiver operating characteristic (ROC) curve was used to define the best point between the sensitivity and specificity.

A test with dichotomous results was employed to ascertain the accuracy, sensitivity, and specificity of the methodology. The results were expressed in two categories: positive and negative. A range of statistical parameters, including sensitivity, specificity, accuracy, positive, and negative predictive values, prevalence, Youden's Index, positive and negative likelihood ratios (+LR and -LR), were verified with EpiDat version 3.1 of January 2006, developed by the Xunta de Galicia and Pan American Health Organization. The adopted significance level was 5% or 0.05.

To verify whether the sample size used in this study was representative, R software v. 4.0.3 was employed, and the effect size, interpreted according to Cohen^(15)^, was calculated in the association analyses based on the Chi-square tests.

## RESULTS

**Figure 1:**
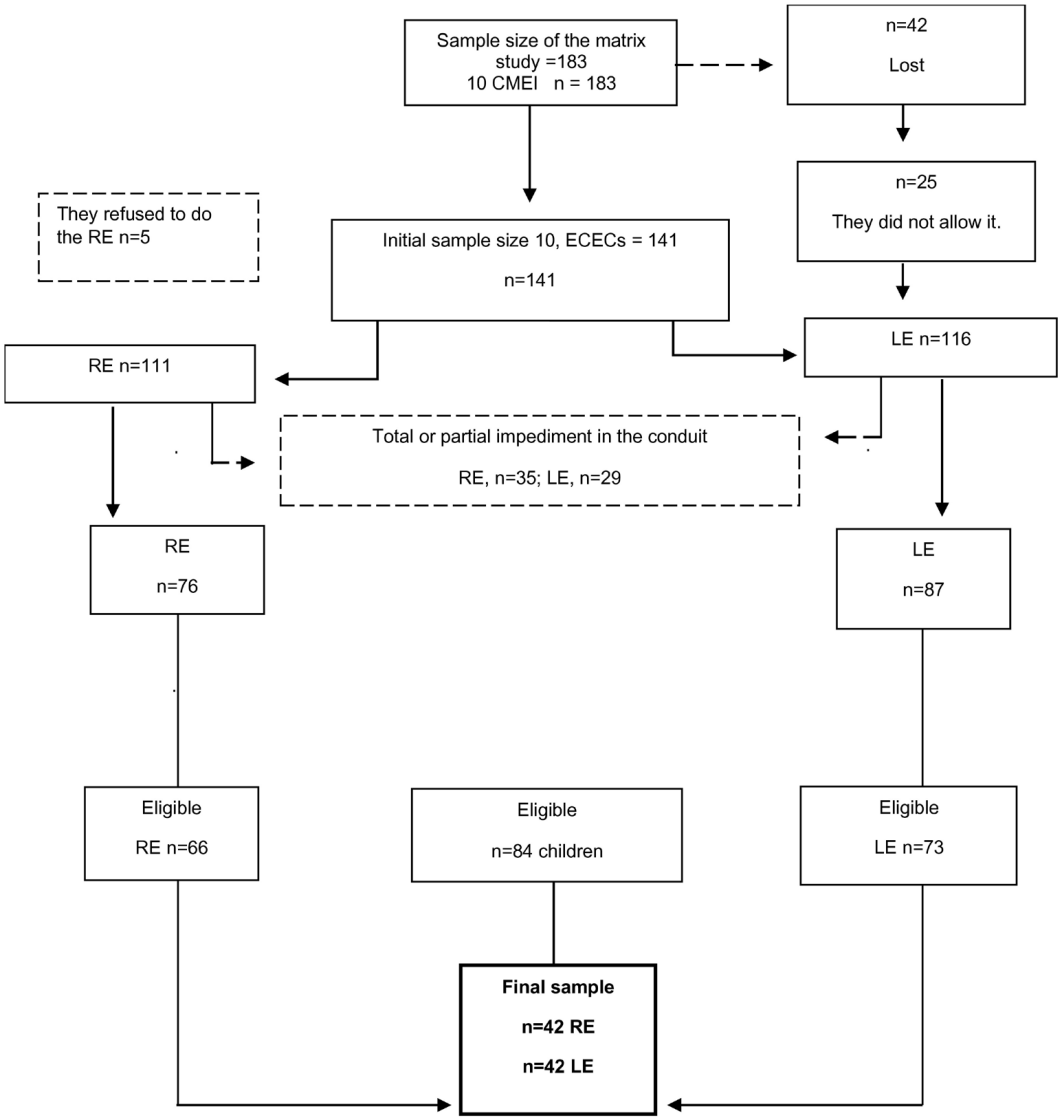
Flowchart of sample selection of children aged 6 to 42 months old in public early public childhood education in Goiânia, Goiás, Brazil, between May and August 2018

The school hearing screening was conducted between May and August 2018. The sample initially comprised 141 children from the matrix project; however, only 84 were eligible, as shown in Figure 1. Children were considered ineligible if they missed school on the day of the collection, refused to participate at the time of the study, had a total or partial cerumen plug, did not complete all the examination, presented tympanograms without a specific configuration and/or records with interference, or had already had known hearing problems.

**Caption:** RE = right ear; LE = left ear; ECECs = Municipal Center for Early Childhood Education Centers

The information provided in Table 1 shows that 53.60% (n=45) of the children were female, with a median age of 24 months and P25 (14) and P75 (35) months of age. The most prevalent economic level (56.0% [n=47]) was class C, which indicates a middle-class income^(14)^. No association was found between the hearing screening examinations and the studied variables.

**Table 1:** Demographic and socioeconomic characteristics and frequency analysis of the group that underwent hearing screening (TEOAEs and tympanometry)

**Characterization**	**Total n (%)**
**Sex (n=84, %)**	
Female	45 (53.60)
**Age in months (n=84, %)**	
Median	24
P25	14
P75	35
**Child's race (n=84, %)**	
White	38 (45.20)
Brown	40 (47.6)
Black	
**Father's race (n=84, %)**	
White	28 (33.30)
Brown	43 (51.20)
Black	13 (15.5)
**Mother's race (n=84, %)**	
White	28 (33.30)
Brown	43 (51.20)
Black	13 (15.5)
**Per capita family income R$**	
Median	733.33
P25	430.80
P75	1000.00
**Economy (INEP) (n=84, %)**	
A-B	31 (36.9)
C	47 (56.0)
D-E	6 (7.1)
**Time at the early childhood education center in months (n=84, %)**	
Median	18
P25	12
P75	24
**Children under five at home (n=81, %)**	
Yes	64 (79.01)
No	17 (20.98)
**Smoking during pregnancy (n=83, %)**	
Yes	2 (2.4)
No	82 (97.6)
**Breastfed at 1 a.m. (n=82, %)**	
Yes	64 (76.2)
No	18 (21.4)
**Use of bottle (n=83, %)**	
Yes	54 (64.3)
No	29 (34.5)
**Previous hearing problem (n=84, %)**	
Yes	10 (11.9)
No	73 (86.90)
Did not know	1 (1.2)
**Transient Evoked Otoacoustic Emissions (n=84, %)**	
RE	66 (78.6)
LE	73 (86.90)
**Tympanometry (n=84, %)**	
RE	66 (78.6)
LE	73 (86.90)

**Caption:** n = number of participants; RE = right ear; LE = left ear; P = percentile; TEOAEs = transient evoked otoacoustic emissions

Initially, we compared the results of children according to age groups: 6–23.99 months and ≥2 years; however, no differences were observed for the study variables in the studied age groups. The average of the minimum noise was 48.60 dB (standard deviation [SD], 5.88 dB; 95% confidence interval [CI] = 46.72–50.47), and the maximum noise was 73.96 dB (SD, 11.39 dB; 95% CI = 70.32–77.60). When Fisher’s exact and Chi-square tests were applied to verify the association of TEOAE results at different cutoff points, an association of TEOAEs was observed at all the cutoff points evaluated, both in the right and left ears (Table 2).

**Table 2:** Comparison of tympanometry and TEOAE results at different cutoff points in 66 right and 73 left ears from 84 children, prior to the random selection of 42 right and 42 left ears for the final database

**n**		**TEOAEs**			***p-value***
		**Refer**		**Pass**	
		**n (%)**		**n (%)**	
		Refer	8 (32.0)	17 (68.0)	0.04*
Peak -100 daPa RE^1^	(66)				
		Pass	4 (9.8)	37 (90.2)	
					
		Refer	6 (60.0)	4 (40.0)	< 0.01*
Peak-150 daPa RE^(1)^	(66)				
		Pass	6 (10.7)	50 (89.3)	
					
		Refer	5 (71.4)	2 (28.6)	0.02*
Peak -200 daPa RE^(1)^	(66)				
		Pass	7 (11.9)	52 (88.1)	
					
		Refer	6 (85.7)	1 (14.3)	<0.01*
Comp. Sta. <0.2 mL RE^(1)^	(66)				
		Pass	6 (10.2)	53 (89.8)	
					
		Refer	11 (36.7)	19 (63.3)	**0.02** *
Peak -100 daPa LE^2^	(73)				
		Pass	3 (7.0)	40 (93.0)	
					
		Refer	7 (43.8)	9 (56.2)	0.01*
Peak -150 daPa LE^(1)^	(73)				
		Pass	7 (12.3)	50 (87.7)	
					
		Refer	6 (50.0)	6 (50.0)	0.01*
Peak -200 daPa LE^(1)^	(73)				
		Pass	8 (13.1)	53 (86.9)	
					
		Refer	5 (71.4)	2 (28.6)	0.01*
Comp. Sta. <0.2 mL LE^(1)^	(73)				
		Pass	9 (13.6)	57 (86.4)	

1 Fisher's exact test;

2 Chi-square;

* Normality values, *p-value*: significance level ≤0.05;

**Caption:** TEOAEs = transient evoked otoacoustic emissions; RE = right ear; LE = left ear; Comp = compliance

The Kappa test (Table 3) showed strong agreement between the right and left ears. Thus, one ear of each of the 84 children was randomly selected to obtain a single bank with 42 right and 42 left ears to determine the accuracy of the test. After creating a single bank with 84 participants, the Student’s t-test was performed to investigate the difference in means of the maximum noise levels during the TEOAE examination. The results demonstrated no significant difference in the maximum noise levels found in the test environment between the groups of children with Refer on the TEOAE examination (M=76.39; SD=13.40) and those with a Pass (M=73.35; SD=10.99) (t [38] = 0.67, p=0.50).

**Table 3:** Interaural agreement in TEOAE, Tympanometry, and Static Compliance Tests in 84 Children (66 Right Ears and 73 Left Ears), evaluated using the Kappa Test

**Test**	**Kappa test**	***p-value***	**Agreement (%)**
TEOAEs	0.809	**<0.001** *	94.54
Comp. Sta.	0.543	0.012*	94.54
-200 daPa	0.446	0.008*	89.00
-150 daPa	0.377	0.016*	83.63
-100 daPa	0.692	<0.001*	85.45

* *p-value,* significance level ≤0.05;

**Caption:** Significance level, p<0.05; TEOAEs = transient evoked otoacoustic emissions; Comp. Sta = static compliance

**Figure 2:**
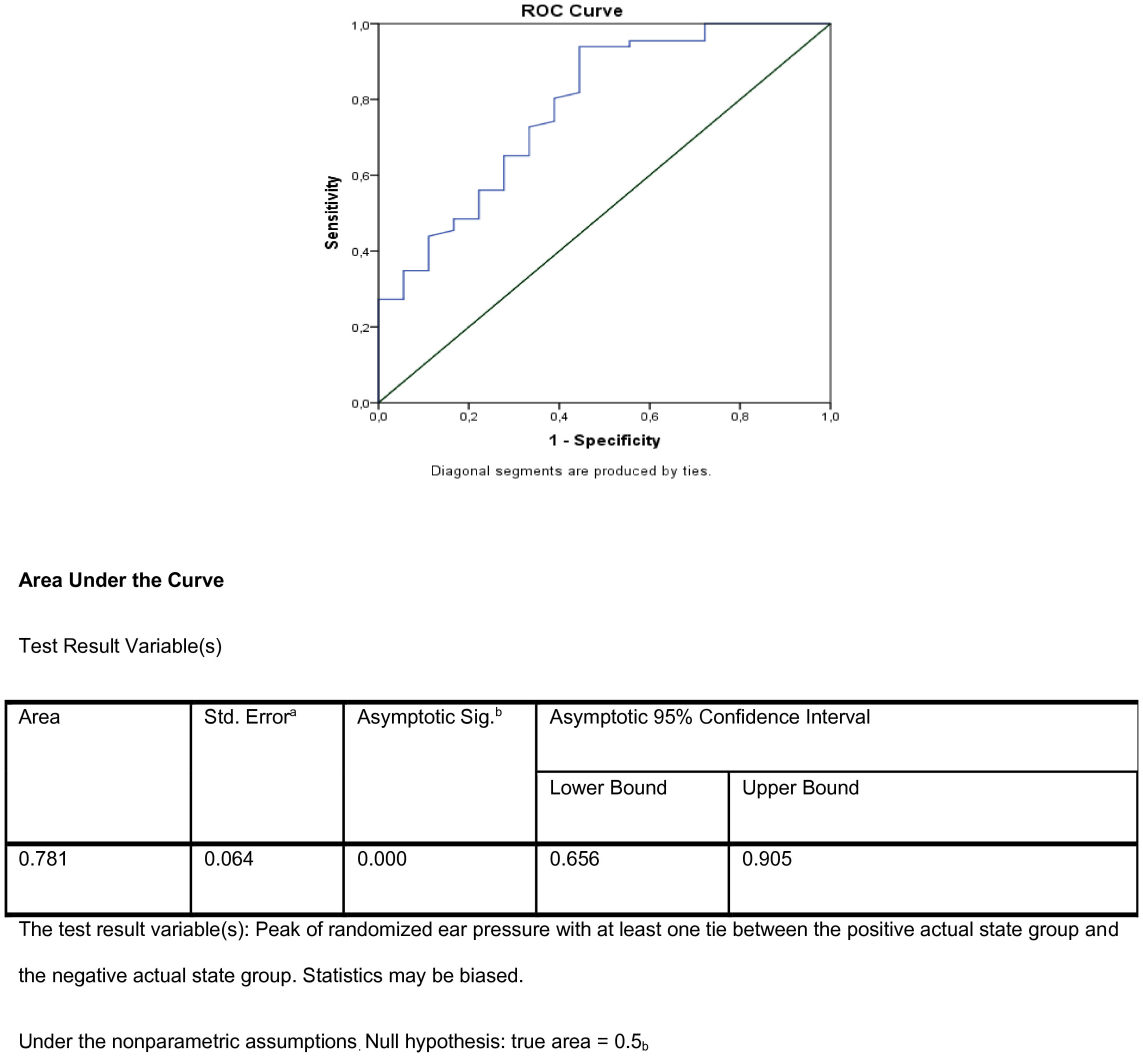
Receiver Operating Characteristic (ROC) curves for the specificity and sensitivity of peak pressure in 84 ears (42 right and 42 left) from 84 public school children who underwent school-based hearing screening

Subsequently, tests to assess the correlation between the variables, static compliance, and TPP found a positive correlation (r=0.325, p=0.003). The constructed ROC curve displayed an area over the curve >70%, which was statistically significant (AUC=0.781, p<0.001; 95% CI = 0.656–0.905). Furthermore, 78.1% of the randomly chosen clinical cases had higher scores than non-clinical cases. The ROC curve was obtained with the numerical values of the pressure peak expressed in daPa in the 84 pooled ears and compared with the TEOAE values. The relationship between the continuous independent variable *y* (pressure values) and the dependent variable *x* (TEOAE test results) is presented in Figure 2.

With the unified sample, the test results were compared with the different TPP (-100 daPa; -150 daPa; -180 daPa; -200 daPa) and static compliance of ≥2 mL, with the TEOAE result. Considering the peak at -200 daPa, the number of Refers reduced, going from 38% (n=32/84) at -100 daPa to 13% (n=11/84) at -200 daPa, and comparing with the static compliance, it reduced to 12% (n=10/84) (Table 4).

**Table 4:** Comparison of tympanometry, static compliance, and TEOAE results at different cutoff points in 84 ears from children in school-based hearing screening

**n**		**TEOAEs**			***p- value***
		**Refer**		**Pass**	
		**(%)**		**n (%)**	
		Refer	12 (37.5)	20 (62.5)	**0.007** *
Peak -100 daPa^2^	(84)				
		Pass	6 (11.5)	46 (88.5)	
					
		Refer	10 (58.8)	7 (41.2)	<0.001*
Peak -150 daPa^1^	(84)				
		Pass	8 (11.9)	59 (88.1)	
					
		Refer	10 (71.4)	4 (28.6)	<0.001*
Peak -180 daPa^(1)^	(84)				
		Pass	8 (11.4)	62 (88.6)	
					
		Refer	8 (72.7)	3 (27.3)	<0.001*
Peak -200 daPa^(1)^	(84)				
		Pass	10 (13.7)	63 (86.3)	
					
		Refer	9 (90.0)	1 (10.0)	<0.001*
Comp Est^(1)^	(84)				
		Pass	9 (12.2)	65 (78.6)	

1 Fisher's exact test;

2 Chi-square test;

* *p- value*: significance level ≤0.05 are shown in bold;

**Caption:** TEOAEs = transient evoked otoacoustic emissions; Normality values, Comp. Sta. = static compliance

The sensitivity, specificity, accuracy, positive predictive and negative predictive values were verified using EpiDat v.3.1. Table 5 also shows the prevalence, Youden index, positive likelihood ratio, and negative likelihood ratio of the cutoff points for the groups (TEOAE Refer and presence of TEOAEs). Static compliance indicated an accuracy of 88.10% (95% CI: 80.57–95.62), specificity of 98.42% (95% CI: 94.78–100.00), and sensitivity of 50.00% (95% CI: 24.12–75.88). The analysis showed that static compliance had greater accuracy and specificity, although with limited sensitivity. For the TPP at –200 daPa, the accuracy was 84.52% (95% CI: 76.19–92.85), specificity was 95.45% (95% CI: 89.67–100.00), and sensitivity was 44.44% (95% CI: 18.71–70.18). However, when evaluating the peak at –180 daPa suggested by the ROC curve (Figure 2), the accuracy was 85.71% (95% CI: 77.64–93.79), the specificity was 93.94% (95% CI: 87.43–100.00), and the sensitivity increased to 55.56% (95% CI: 29.82–81.29). Positive and negative predictive values were also higher at this cutoff, reaching 71.43% (95% CI: 44.19–98.66) and 88.57% (95% CI: 80.40–96.74), respectively. The Youden index was 0.49, the positive likelihood ratio was +9.17, and the negative likelihood ratio was -0.47 (Table 5).

**Table 5:** Diagnostic accuracy of tympanometry and static compliance at different cutoff points relative to TEOAE results in 84 ears

**Cutoff point**	**Value**	**95% CI**
**Static compliance**		
Sensitivity (%)	50.00	24.12–75.88
Specificity (%)	98.48	94.78–100.00
Accuracy (%)	88.10	80.57–95.62
Positive predictive value (%)	90.00	66.41–100.00
Negative predictive value (%)	87.84	79.72–95.96
Prevalence (%)	21.43	12.06–30.80
Youden index	0.48	0.25–0.72
Positive likelihood ratio	33.00	4.47–243.64
Negative likelihood ratio	0.51	0.32–0.81
**Cutoff point -100 daPa**		
Sensitivity (%)	66.67	42.11–91.22
Specificity (%)	69.70	57.85–81.54
Accuracy (%)	69.05	58.57–79.53
Positive predictive value (%)	37.50	19.16–55.84
Negative predictive value (%)	88.46	78.82–98.11
Prevalence (%)	21.43	12.06–30.80
Youden index	0.36	0.12–0.61
Positive likelihood ratio	2.20	1.35–3.59
Negative likelihood ratio	0.48	0.24–0.94
**Cutoff point -150 daPa**		
Sensitivity (%)	55.56	29.82–81.29
Specificity (%)	89.39	81.21–97.58
Accuracy (%)	82.14	73.36–90.93
Positive predictive value (%)	58.82	32.49–85.16
Negative predictive value (%)	88.06	79.55–96.57
Prevalence (%)	21.43	12.06–30.80
Youden index	0.45	0.21–0.69
Positive likelihood ratio	5.24	2.32–11.81
Negative likelihood ratio	0.50	0.29–0.84
**Cutoff Point -180 daPa**		
Sensitivity (%)	55.56	29.82–81.29
Specificity (%)	93.94	87.43–100.00
Accuracy (%)	85.71	77.64–93.79
Positive predictive value (%)	71.43	44.19–98.66
Negative predictive value (%)	88.57	80.40–96.74
Prevalence (%)	21.43	12.06–30.80
Youden index	0.49	0.26–0.73
Positive likelihood ratio	9.17	3.25–25.83
Negative likelihood ratio	0.47	0.28–0.80
**Cutoff Point -200 daPa**		
Sensitivity (%)	44.44	18.71–70.18
Specificity (%)	95.45	89.67–100.00
Accuracy (%)	84.52	76.19–92.85
Positive predictive value (%)	72.73	41.86–100.00
Negative predictive value (%)	86.30	77.73–94.87
Prevalence (%)	21.43	12.06–30.80
Youden index	0.40	0.16–0.63
Positive likelihood ratio	9.78	2.89–33.13
Negative likelihood ratio	0.58	0.38–0.88

**Caption:** 95% CI = 95% Confidence interval

## DISCUSSION

A comparative analysis of the cutoff points of TPP and static compliance volume was carried out, considering the TEOAE results of 84 children (42 right and 42 left ears), 6–42 months of age and enrolled in public early childhood education in the city of Goiânia, Goiás, Brazil. This study aimed to verify, with greater accuracy, the cutoff points of TPP capable of showing tympanometry Refer associated with TEOAE Refer to identify any hearing alteration. The most negative values of the TPP generated greater agreement with the TEOAE results. A new cutoff point of -180 daPa was suggested based on the ROC curve, offering greater precision and a higher Youden index. However, it presented low sensitivity, with TEOAE results used as the reference variable, as confirmed by the EpiDat software (version 3.1).

No significant differences were observed regarding age or sex. Altered tympanometry results were associated with TEOAE Refer, suggesting the presence of middle ear dysfunction in this asymptomatic population. Additionally, a significant correlation was identified between static compliance and TPP values.

Given the assumption that middle ear dysfunction prevents the detection of TEOAEs, it is hypothesized that the absence of TEOAEs, when combined with tympanometric alterations and/or reduced static compliance, reflects middle ear pathology^(3)^. However, in children under three years old, a static compliance of 0.6 mL has not been consistently associated with middle ear effusion, reinforcing recommendations against relying solely on tympanometry for diagnosis^(16)^. In the present study, static compliance values ranged from the 25^th^ percentile (0.2 mL) to the 75^th^ percentile (0.5 mL), with a median of 0.4 mL.

Minimal hearing loss can negatively affect speech development^(17)^. Nonetheless, many children with mild hearing loss may pass hearing screenings and Refer to receive appropriate follow-up. Mild hearing loss may precede progressive or late-onset hearing loss, and its impact is exacerbated when combined with other developmental conditions.

Previous studies have demonstrated that a history of recurrent otitis media with effusion can interfere with TEOAE generation and transmission^(18)^. Furthermore, it can affect long-latency auditory evoked potentials in children who underwent myringotomy, leading to long-term auditory processing deficits^(19)^.

Both behavioral auditory assessments and electrophysiological measurements have evidenced such negative auditory outcomes^(20)^. Otitis media with effusion has also been shown to impact P300 responses and auditory behavioral performance in different pediatric populations^(21)^.

Individual test results should not be interpreted in isolation. In this study, the combined analysis of TPP, static compliance, and TEOAEs highlighted the importance of an integrated diagnostic approach. For example, a type C tympanometric curve reflects eustachian tube dysfunction—typically a transient condition^(22)^. However, during the screening, it is impossible to determine whether the child is in the onset, progression, or resolution phase of a middle ear condition. In such cases, TEOAE Refer may serve as an additional indicator to confirm the presence of middle ear dysfunction.

TEOAEs are a valuable tool for screening populations at high risk for recurrent otitis media. Nevertheless, one limitation can be high levels of biological noise in infants. The equipment's microphone captures both environmental noise and the child's own physiological noise^(23)^. The test proceeds only when the noise level does not exceed the stimulus level; however, internal noise generated by young children can contribute to TEOAE Refer.

Spontaneous otoacoustic emissions are low-amplitude signals returning from the cochlea to the external auditory canal. A low noise floor is essential to ensure valid TEOAE recordings. Reliable TEOAE results depend on testing in quiet environments, with minimal external noise and vibrations. Proper probe selection and placement, ensuring a good acoustic seal, substantially reduces background noise^(6)^. Despite suboptimal environmental conditions in the present study, careful probe placement mitigated external noise interference. No significant difference in environmental noise levels was observed between children who received Pass and those who received Refer on the TEOAEs.

In a previous study involving two groups—one with a history of myringotomy with tube insertion and another without middle ear effusion—sensitivity and specificity measures were used alongside static compliance, gradient, and acoustic reflex testing to validate otoscopic findings and inform surgical decisions^(17)^. The present study evaluated TPP and static compliance cutoff points against TEOAE outcomes. The literature contains different tympanometric cutoff points associated with hearing impairment. TEOAEs have been widely adopted in preschool and school-age hearing screening as a fast and objective alternative when conventional audiometry—the gold standard—is not feasible^(7)^. Children who pass the TEOAE screening have a lower likelihood of hearing loss at the time of testing, supporting its relevance for use in underserved communities^(24)^.

The target population of this study was children enrolled in public daycare centers. A previous study conducted in the Brazilian population reported absent TEOAEs in 20% of children 1 to 5 years old, while tympanometry showed abnormalities in 30% of the same group^(12)^. Similar findings were observed in the present study, with a TEOAE Refer rate of 21.43% (n = 18). However, tympanometry Refer rates depend on the cutoff point adopted. When considering a TPP of -100 daPa, the tympanometry Refer rate was 38.10% (n = 32), but this decreased to 16.66% (n = 14) when the cutoff was adjusted to -180 daPa, as suggested by the ROC curve.

A similar study with preschool-age children reported a TEOAE test sensitivity of 1.00 (95% CI: 0.054–1.00) and specificity of 94% (95% CI: 0.88–0.97)^(24)^. In the present study, the tympanometry cutoff point that achieved the highest accuracy when compared with TEOAEs was -180 daPa, with an accuracy of 85.71% (95% CI: 77.64–93.00), a sensitivity of 55.96% (95% CI: 29.82–81.29), and a specificity of 93.94% (95% CI: 83.43–100.00). For static compliance, specificity increased to 98.48% (95% CI: 94.78–100.00), although sensitivity decreased to 50% (95% CI: 24.12–75.88).

A study involving children aged 5 to 10 years in Brazil reported a tympanometry pass rate^(25)^ comparable to that observed in the present study when using a TPP cutoff of –100 daPa. Similar trends were found for TEOAE results and cases that passed both tests. When the TPP cutoff was adjusted to –200 daPa, an increase in the overall pass rate was observed, which was also reflected in the analysis based on static compliance. The similarity between studies at the –100 daPa cutoff may be attributable to the cutoff value itself. Notably, when applying the –180 daPa cutoff proposed in the present study, the pass rate remained consistent, with a modest increase in the proportion of true positives compared to the more negative cutoff.

An analysis of 142 tympanograms found that values as negative as -150 daPa were not indicative of middle ear effusion. No significant correlation was identified between tympanometric curves and middle ear effusion^(13)^, except in flat type B curves, which were associated with effusion in 90% of cases. However, tympanometric parameters should not be interpreted in isolation, as negative TPP values may occur in ears without effusion. In another study, 80.2% were diagnosed with middle ear effusion with flat tympanograms^(16)^.

Tympanometric evaluations were performed during both symptomatic and asymptomatic visits, resulting in 4,246 examinations, of which 76% were classified as Pass and 24% as Refer (either inconclusive or indicating dysfunction). Most tympanograms classified as types A, C1, and C2 were associated with normal middle ear status. Although type B tympanograms were generally associated with middle ear effusion, no definitive correlation was established, as this curve type did not consistently differentiate between ears with and without effusion^(26)^.

In a study of 515 children (mean age 16 months; range 6–35 months), tympanometric findings were compared with pneumatic otoscopy results^(26)^. Evaluations were conducted using a 266 Hz probe tone^(9)^ and the Jerger classification system^(9-11)^, later modified by Orchik et al.^(13)^ and Zielhuis et al.^(27)^. Tympanograms were categorized based on TPP values: type A for TPP up to -100 daPa, type C1 for TPP between -100 and -200 daPa, type C2 for TPP below -200 daPa, and type Cs for reduced compliance and amplitude.-In the present study, Refer rates were lower when applying the cutoff point of -180 daPa compared with TPP thresholds of -100 daPa and -150 daPa.

Tympanometry used in isolation has limited diagnostic value, as it cannot distinguish between different types of otitis. Nevertheless, a type A tympanogram generally indicates normal middle ear function, while a type B tympanogram suggests effusion^(27)^. Tympanometry enhances diagnostic accuracy and serves as an objective adjunct to confirm or rule out middle ear effusion in cases where pneumatic otoscopy yields inconclusive results. Although tympanometric values alone are insufficient for differentiating otitis media with effusion (OME), combining them with TEOAEs significantly improves diagnostic accuracy^(28)^. This association often leads to Refer classifications and medical referrals during hearing screening based on the presumption of a link between tympanometric patterns and effusion^(13)^.

However, type C tympanograms could present diagnostic challenges, as they may reflect either a transient eustachian tube dysfunction (without effusion) or an early/late stage of middle ear effusion^(29)^. Furthermore, mild eustachian tube dysfunction might not be detected by tympanometry^(30)^.

Determining an appropriate cutoff point supports clinical decision-making, allowing more accurate classification between Pass and Refer outcomes. Validation of the proposed cutoff point should involve multiple strategies, including sensitivity analyses and the use of independent datasets to mitigate bias and minimize the risk of type I errors. The Youden index is particularly useful in identifying the optimal balance between true positives and true negatives^(31)^. This study evaluated multiple TPP peaks, and -180 daPa demonstrated the highest sensitivity, accuracy, and Youden index.

While the TPP cutoff of -200 daPa^(1,5,6)^ has been referenced in previous studies involving infants, the present study aimed to assess the diagnostic accuracy of various TPP thresholds reported in the literature among Brazilian children. The sensitivity, accuracy, negative predictive value, and Youden index were all lower with the -200 daPa threshold than with the -180 daPa. Notably, the prevalence of Refer was equivalent at both -180 daPa and -200 daPa.

However, lower sensitivity may lead to false negatives, i.e., cases in which a hearing alteration is not identified during screening. This is undoubtedly a critical point in any screening process, as individuals who have alterations may not be properly referred.

The low sensitivity (55.6%) of the cutoff point at -180 daPa is an aspect that requires critical discussion, especially due to the risk of false negatives in clinical and screening contexts. The adoption of a less rigid limit, such as -100 daPa, increases sensitivity, but at the expense of excessive false positives and consequent unnecessary referrals. Although the stricter limit of -200 daPa raises the specificity, it accentuates the loss of real cases, with risk of misinterpretations as absence of the “disease.” In this scenario, the evaluation of accuracy becomes fundamental to help select the parameter. Thus, the cutoff point at -180 daPa, although presenting a sensitivity limitation, represents the best balance between sensitivity, specificity, and accuracy, making it the most appropriate value for child hearing screening programs. The cutoff point was defined with the aim of greater specificity and a lower rate of false positives, a common strategy in population screenings to avoid unnecessary referrals, that is, fewer healthy children being rated as altered. According to the British NHS guidelines^(32)^, implementation of a continuous surveillance system facilitates the detection of cases that may not be identified in the initial screening, especially in public or school programs.

Certain limitations of the present study must be considered. The absence of a single equipment that can simultaneously perform TEOAEs and tympanometry during screening in a school environment may be a methodological restriction. In addition, the sample size, restricted to a regional population and a single equipment model, may limit the generalization of the results to other contexts.

Despite these limitations, the findings reinforce the clinical utility of the proposed cutoff point. To broaden its applicability, future research should include larger samples, different age groups, multicenter studies, comparison with other screening tools (e.g., ABR – Auditory Brainstem Response), and longitudinal designs to monitor the evolution of middle ear changes and their impact on auditory development. Developing integrated equipment that performs tympanometry, TEOAE, and automated ABR in a school environment could also increase the efficiency of school hearing screening programs. Finally, the analysis of additional tympanometry parameters, such as the gradient, and the evaluation of the cost-effectiveness of these cutoff points could refine diagnostic accuracy and optimize public health strategies.

## CONCLUSION

The cutoff point of -180 daPa was shown to improve the accuracy of tympanometry in pediatric hearing screening associated with compliance ≥2 mL, reducing unnecessary referrals. However, it does not eliminate the risk of false negatives, emphasizing the need for its association with continuous surveillance strategies. These findings reinforce the adoption of evidence-based tympanometric parameters adapted to pediatric populations, which can strengthen screening protocols and help improve hearing health care.
